# Circulating let-7e-5p, miR-106a-5p, miR-28-3p, and miR-542-5p as a Promising microRNA Signature for the Detection of Colorectal Cancer

**DOI:** 10.3390/cancers13071493

**Published:** 2021-03-24

**Authors:** Camila Meirelles S. Silva, Mateus C. Barros-Filho, Deysi Viviana T. Wong, Julia Bette H. Mello, Livia Maria S. Nobre, Carlos Wagner S. Wanderley, Larisse T. Lucetti, Heitor A. Muniz, Igor Kenned D. Paiva, Hellen Kuasne, Daniel Paula P. Ferreira, Maria Perpétuo S. S. Cunha, Carlos G. Hirth, Paulo Goberlânio B. Silva, Rosane O. Sant’Ana, Marcellus Henrique L. P. Souza, Josiane S. Quetz, Silvia R. Rogatto, Roberto César P. Lima-Junior

**Affiliations:** 1 Department of Physiology and Pharmacology, Faculty of Medicine, Federal University of Ceará, Fortaleza 60430-270, Brazil; liv.nobre@alu.ufc.br (L.M.S.N.); cwswanderley@gmail.com (C.W.S.W.); larisse.lucetti@fiocruz.br (L.T.L.); heitorico@hotmail.com (H.A.M.); igorkenned10@gmail.com (I.K.D.P.); 2 International Research Center—CIPE, A.C. Camargo Cancer Center, Sao Paulo 01525-001, Brazil; mfilho@accamargo.org.br (M.C.B.-F.); hellen.kuasne@mcgill.ca (H.K.); 3 Department of Head and Neck Surgery, Hospital das Clínicas da Faculdade de Medicina da Universidade de São Paulo/LIM-28-São Paulo, Sao Paulo 05403-000, Brazil; 4 Department of Pathology and Forensic Medicine, Faculty of Medicine, Federal University of Ceará, Fortaleza 60430-160, Brazil; deysiviviana@ufc.br or; 5 Haroldo Juaçaba Hospital—Cancer Institute of Ceará, Fortaleza 60430-230, Brazil; pathopatty@hotmail.com (M.P.S.S.C.); carlos.hirth@ufc.br (C.G.H.); paulo.silva@icc.org.br (P.G.B.S.); rosane@unifor.br (R.O.S.); josiane.quetz@unifenas.br (J.S.Q.); 6 Molecular Carcinogenesis Program, Brazilian National Cancer Institute (INCA), Rio de Janeiro 20230-240, Brazil; juliahmello@gmail.com; 7 Cesar Cals Hospital, Fortaleza 60015-152, Brazil; danpaulapessoa@gmail.com; 8 School of Medicine, University of Fortaleza, Fortaleza 60811-905, Brazil; 9 Department of Clinical Medicine, Faculty of Medicine, Federal University of Ceará, Fortaleza 60430-160, Brazil; souzamar@ufc.br; 10 Department of Clinical Genetics, University Hospital of Southern Denmark, 7100 Vejle, Denmark; 11 Institute of Regional Health Research, University of Southern Denmark, 5000 Odense, Denmark; 12 Danish Colorectal Cancer Center South, 7100 Vejle, Denmark

**Keywords:** colorectal cancer, blood, microRNA, diagnosis

## Abstract

**Simple Summary:**

The detection of early-stage colorectal cancer increases the chance to prevent tumor progression and death by the disease. Colonoscopy is one sensitive screening test to detect malignant or potentially malignant lesions in the intestines. However, it has some disadvantages, including sedation requirements, increased risk of colon perforation, and bleeding. Circulating microRNAs (miRNAs) in plasma or serum from cancer patients have been investigated and described as potential diagnostic or prognostic markers. We conducted an miRNAs screening test in plasma samples from colorectal cancer patients and subjects without cancer, aiming to identify markers for the early detection of the disease. We identified and validated four miRNAs capable of distinguishing cancer from non-cancer cases. Our non-invasive diagnostic biomarkers presented high performance and are easily applicable to clinical practice.

**Abstract:**

Colorectal cancer (CRC) is a disease with high incidence and mortality. Colonoscopy is a gold standard among tests used for CRC traceability. However, serious complications, such as colon perforation, may occur. Non-invasive diagnostic procedures are an unmet need. We aimed to identify a plasma microRNA (miRNA) signature for CRC detection. Plasma samples were obtained from subjects (*n* = 109) at different stages of colorectal carcinogenesis. The patients were stratified into a non-cancer (27 healthy volunteers, 17 patients with hyperplastic polyps, 24 with adenomas), and a cancer group (20 CRC and 21 metastatic CRC). miRNAs (381) were screened by TaqMan Low-Density Array. A classifier based on four differentially expressed miRNAs (miR-28-3p, let-7e-5p, miR-106a-5p, and miR-542-5p) was able to discriminate cancer versus non-cancer cases. The overexpression of these miRNAs was confirmed by RT-qPCR, and a cross-study validation step was implemented using eight data series retrieved from Gene Expression Omnibus (GEO). In addition, another external data validation using CRC surgical specimens from The Cancer Genome Atlas (TCGA) was carried out. The predictive model’s performance in the validation set was 76.5% accuracy, 59.4% sensitivity, and 86.8% specificity (area under the curve, AUC = 0.716). The employment of our model in the independent publicly available datasets confirmed a good discrimination performance in five of eight datasets (median AUC = 0.823). Applying this algorithm to the TCGA cohort, we found 99.5% accuracy, 99.7% sensitivity, and 90.9% specificity (AUC = 0.998) when the model was applied to solid colorectal tissues. Overall, we suggest a novel signature of four circulating miRNAs, i.e., miR-28-3p, let-7e-5p, miR-106a-5p, and miR-542-5p, as a predictive tool for the detection of CRC.

## 1. Introduction

Colorectal cancer (CRC) is the third most frequent type of cancer worldwide [1]. CRC is frequently diagnosed at an advanced stage, and distant metastases contribute to its high mortality rate [2]. It is a complex disease that involves interactions between genetic and environmental factors in the intestinal epithelium [3]. The normal epithelium accumulates changes over 20–40 years, progressing from different dysplasia grades to the establishment of local and distant metastasis [4,5]. Remarkably, the long-term tumor development opens the perspective for early disease traceability [6]. 

Colonoscopy is a gold-standard screening method that significantly reduces the mortality rate since it allows the detection of precancerous polyps and early-stage CRC. Despite being an outstanding screening tool, several limitations have been described, such as colon perforation, bleeding, sedation requirements, cost, and invasiveness of the procedure [7]. The development of rapid, less invasive, and low-risk procedures complementary to colonoscopy is highly welcome. The fecal occult blood test (FOBT)-based screening, a non-invasive technique, can discover the existence of polyps, adenomas, and tumors in the intestine. However, the FOBT is limited by its reduced positive predictive value [8]. Non-invasive protocols clinically useful in CRC screening programs showing high performance are required.

Blood biomarkers are a promising alternative diagnostic approach to detect CRC [9,10]. The carcinoembryonic antigen (CEA) has been widely used as a blood-based molecular marker for detecting tumor recurrence [11]. Other circulating biomarkers have also been considered for post-operative CRC surveillance, such as cancer antigen 19-9 (CA19-9), cancer antigen 125 (CA125), and Septin 9 methylated DNA [9,12]. Despite being useful for patient monitoring, these markers are also associated with non-neoplastic conditions, such as inflammatory bowel disease and endometriosis, and other types of cancer, including ovarian, gastric, pancreatic, and lung cancer [9]. Notwithstanding, no circulating biomarker is currently available for the early detection of CRC. Circulating microRNAs (miRNAs) have been considered new promising biological markers for cancer detection [10]. 

miRNAs are small non-coding RNAs of 21–25 nucleotides, which repress target messenger RNAs (mRNAs). miRNAs play a critical role in cell signaling networks, and their expression is associated with several tumor types, including colorectal, breast, gastric, lung cancers and sarcomas [13,14,15]. Pathological conditions can impact the miRNA profile, and this is the basis for obtaining liquid biopsies. A liquid biopsy can be obtained at all stages of cancer diagnosis and treatment, allowing non-invasive and real-time monitoring of disease development [16,17]. 

Although many studies propose detecting miRNA signatures for early CRC diagnosis [18,19,20,21,22,23,24], several limitations are observed, such as reduced coverage for miRNA screening [19,22], variable signatures for different disease stages [24], absence of non-cancer groups [20], or lack of data validation. Additionally, some of these studies analyzed only one miRNA as a biomarker [25], miRNA allied with inflammatory mediators [23], or miRNA expression without testing the signature’s power [18,21,26,27,28]. Indeed, these strategies have reduced validity due to tumor heterogeneity and potential lack of specificity. A broader miRNA panel for identifying a suitable signature to be tested in all tumor development stages and validated with gene databases seems an appropriate approach to overcome such limitations and increase diagnostic accuracy, specificity, and sensitivity.

In the present study, plasma samples of healthy subjects and patients at different stages of colorectal carcinogenesis were assessed for identifying an miRNA signature capable of distinguishing cancer patients from those without the disease.

## 2. Results

**Figure 1:**
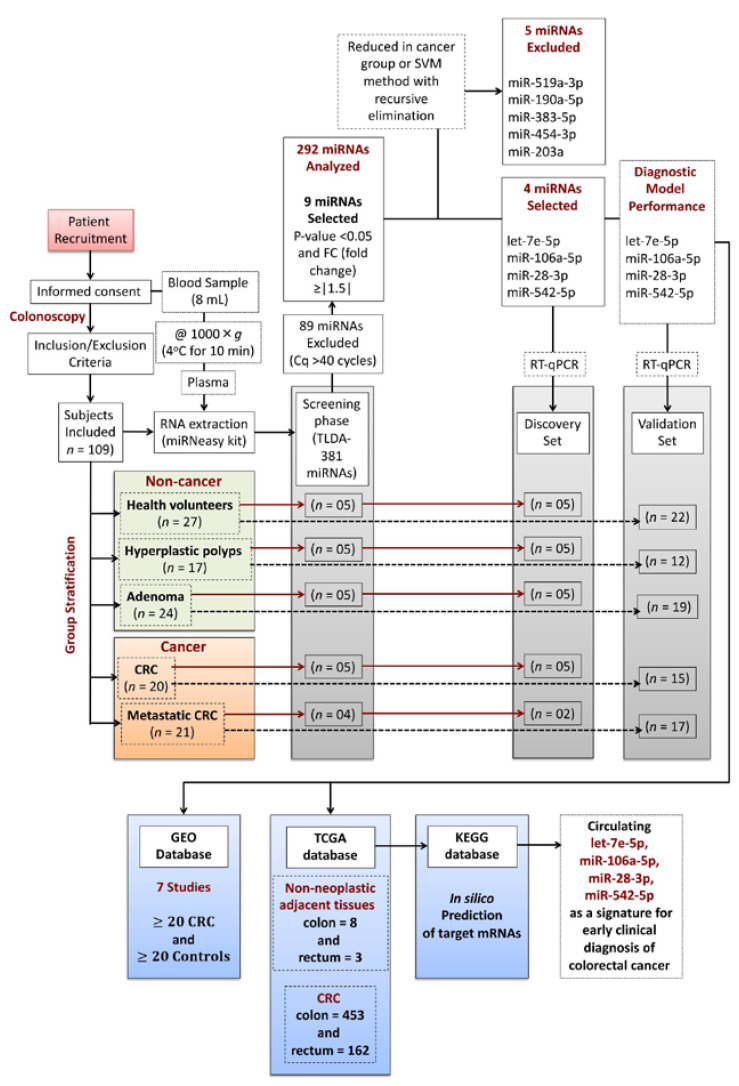
Study workflow to identify a diagnostic miRNA signature from plasma samples of patients at different stages of CRC development. CRC, colorectal cancer.

The clinical–demographic characteristics of subjects included in the study are shown in Table 1. Figure 1 depicts the study workflow designed to identify a diagnostic miRNA signature from plasma samples of patients at different CRC development stages. One hundred and nine subjects (61 female and 48 male) were recruited and stratified into two groups: a non-cancer group (*n* = 68) composed of healthy controls, individuals with hyperplastic polyps, or adenoma and a cancer group (*n* = 41), which included CRC patients and subjects with metastatic CRC (Table 1 and Figure 1). Most of the patients never smoked (66.06%) and used no medications at the recruitment time (54.12%). Antihypertensives, proton-pump inhibitors, and analgesics were drugs reported by the subjects with current medication use.

### 2.1. Circulating miRNA Profile in the Plasma of CRC and Non-CRC Subjects 

A total of 292 out of 381 potential markers was included in the screening phase of our study. Eighty-nine candidates showing constant deficient expression (a null cycle quantification after 40 cycles of amplification) in cancer and non-cancer samples were excluded (Figure 1 and Table S1).

**Figure 2:**
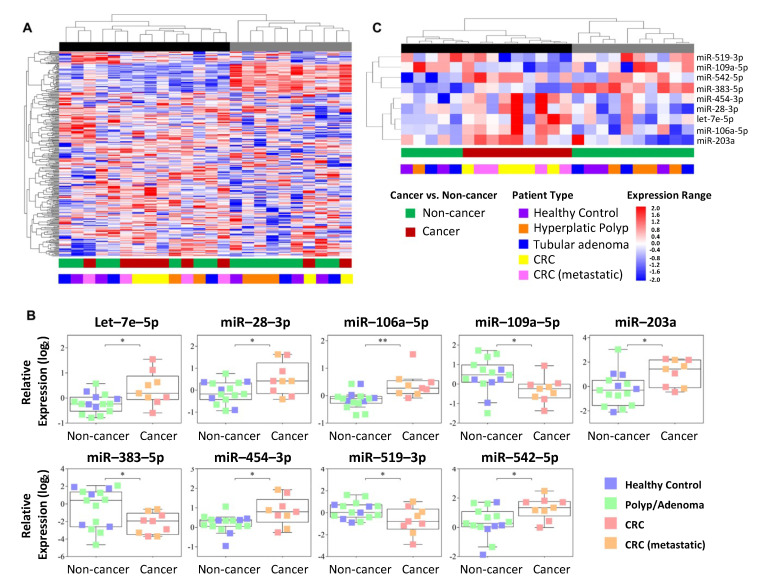
Hierarchical clustering analysis and plots representing plasma miRNAs. (**A**) Unsupervised hierarchical clustering analysis of 292 circulating miRNAs (RT-qPCR-TaqMan Low-Density Array (TLDA) assay). (**B**) Differential expression of nine miRNAs in the plasma of cancer versus non-cancer cases. The boxplot displays the first quartile, median, and third quartiles (interquartile range) and the minimum and maximum values excluding outliers of the log_2_-normalized relative quantification of the miRNAs in plasma (RT-qPCR-TLDA assay). (**C**) Supervised hierarchical clustering analysis comprising the nine differentially expressed miRNAs. The dendrogram demonstrates a stratification of samples into two clusters (black and gray) associated with the cancer status. The lines in the heatmaps represent individual miRNAs, and the columns represent each sample; * *p* < 0.05; ** *p* < 0.01 (*t*-test).

Unsupervised hierarchical clustering analysis did not demonstrate a clear stratification between cancer and non-cancer samples (Figure 2A). These groups were statistically compared, unveiling nine miRNAs with differential expression in plasma (Figure 2B and Table S2). Increased expression levels of miR-542-5p, miR-28-3p, miR-106a-5p, let-7e-5p, miR-454-3p, and miR-203a and decreased expression levels of miR-190a-5p, miR-383-5p, and miR-519a-3p were detected in CRC patients compared with the non-cancer group (Figure 2B). A supervised hierarchical clustering analysis, including these nine miRNAs, revealed a group of six miRNAs associated with cancer- (Figure 2C). One cluster enriched of cancer patients (black) and another (gray) composed exclusively of non-cancer individuals (healthy volunteers and patients with hyperplastic polyps and adenomas) were also observed (top of Figure 2A,C).

### 2.2. Circulating miRNA-Based Model to Predict Colorectal Malignancy

**Figure 3:**
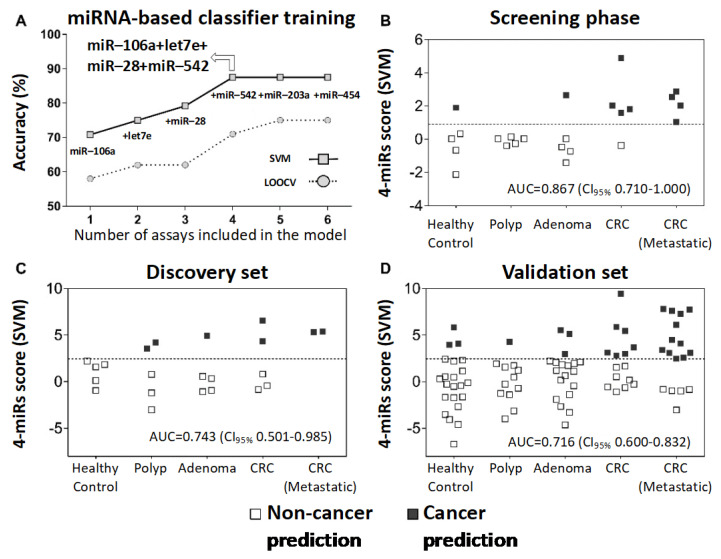
Training and validation of the circulating miRNA-based diagnostic classifier. (**A**) Cancer prediction models including one to six miRNAs (selected by the recursive elimination method) previously detected at higher levels in the blood samples of CRC patients. Representative graphs of overall yield accuracy and LOOCV estimative. (**B**) Application of the four-miR-based classifier (miR-106a + let-7e + miR-28 + miR-542) in the screening phase (evaluated by the TLDA assay). (**C**) The four-miR-based classifier applied to a subset of cases of the discovery set (screening phase) using individual RT-qPCR assays. (**D**) Application of the four-miR-based classifier to a group of samples independent of the screening phase (validation set) using individual RT-qPCR assays. The dotted line indicates the threshold above which a malignant status would be predicted. SVM: support vector machine; LOOCV: leave-one-out cross-validation; AUC: area under the ROC curve; CI_95%_: 95% confidence interval.

A potential diagnostic tool was designed based exclusively on the over-represented circulating miRNAs (*n* = 6) in CRC patients from the discovery set. Six models were tested, including one to six miRNAs (support vector machine (SVM) method with recursive elimination), where a combination of four to six miRNAs achieved the best overall accuracy (87.5%) (Figure 3A). Accordingly, we carried on with the four-miRNAs combination that required fewer assays. The application of the four-miR-based classifier in the screening phase (score = let-7e-5p × 1.037 + miR-106a-5p × 0.9 + miR-28-3p × 0.247 + miR-542-5p × 0.903; cancer prediction threshold >1.024) yielded an 88.9% sensitivity and 86.7% specificity (55.6% and 80.0% in the leave-one-out cross-validation (LOOCV), respectively) (Figure 3B and Table 2). 

### 2.3. Validation of the Circulating miRNAs as a Diagnostic Model

Four putative plasma markers (let-7e-5p, miR-106a-5p, miR-28-3p, and miR-542-5p) and two carefully selected endogenous reference miRNAs (mir-423-5p and mir-361-5p) were further tested using RT-qPCR assays. The sequences of the endogenous and target miRNAs are described in Table S3. This step was carried out in a subset of samples previously analyzed in the screening phase (discovery set; *n* = 22) and validation set (*n* = 85). The same mathematical model previously designed was adopted to support the predictive model performance, adjusting the threshold to achieve the best overall accuracy (cancer prediction threshold >2.442). The method demonstrated a similar classification performance in the discovery (72.7% accuracy, 57.1% sensitivity, 80% specificity, AUC = 0.743) and validation sets (76.5% accuracy, 59.4% sensitivity, 86.8% specificity, AUC = 0.716) (Figure 3C,D and Table 2). The combined four-miR-based classifier had a higher AUC than any single miRNAs marker in the discovery and validation sets (Figure S1).

### 2.4. Performance of the Diagnostic Model in External Datasets of Liquid Biopsies and Solid Tissues

**Figure 4:**
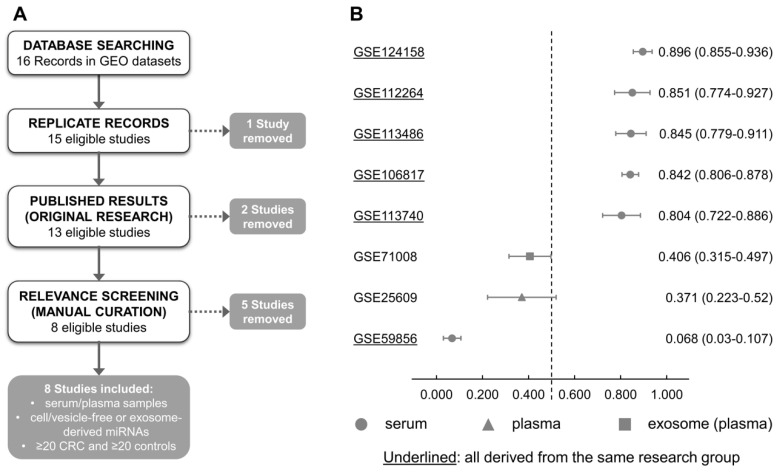
Performance of the four-miR classifier tested in the Gene Expression Omnibus (GEO) dataset. (**A**) Database searching, inclusion and exclusion criteria. (**B**) Among 16 studies found in the GEO datasets, 7 fulfilled the criteria of number of samples (≥20 samples of both CRC and controls), 5 used serum samples and validated our four-miR classifier model, and 3 datasets (exosome, serum, and plasma samples) showed no significant association.

To confirm the performance of our circulating miRNA model, we investigated publicly available databases comprising small non-coding RNAs analysis of liquid biopsy samples from CRC and controls in the Gene Expression Omnibus (GEO). Sixteen data series were found, and seven were included after employing the inclusion/exclusion criteria and curation of the published articles (Figure 4A and Table S4).

This cross-study validation step included five studies assessing small non-coding RNAs from serum (GSE106817, GSE113740, GSE112264, GSE124158, GSE113486, and GSE59856), one from plasma (GSE25609), and one from plasma-derived extracellular vesicles (GSE71008). The available processed values (microarray and high-throughput sequencing) were used to generate the four-miRNA score, and the ROC curve for all studies was assessed. The AUCs varied largely, ranging from 0.068 to 0.896 in different studies, with a median of 0.823 (Figure 4B).

**Figure 5:**
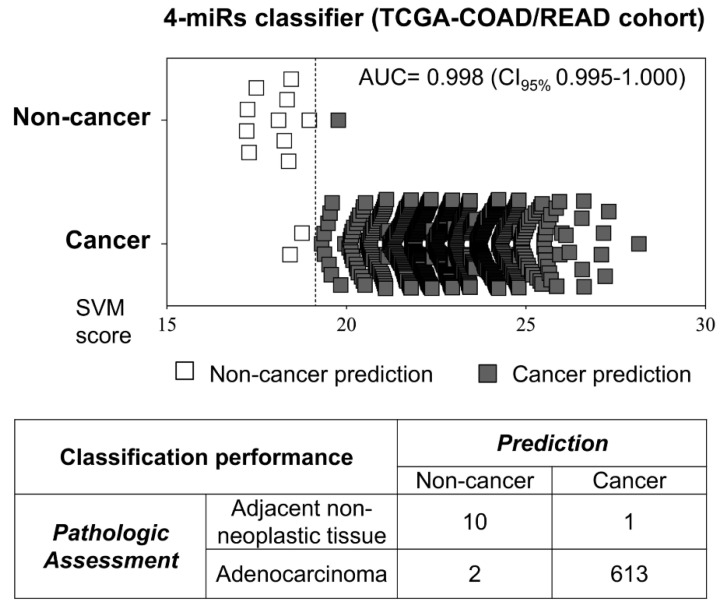
Performance of the four-miR classifier tested in TCGA colorectal primary tumors and adjacent non-cancer tissues. The classifier designed to be a liquid biopsy method also demonstrated high power in discriminating cancer and non-cancer colorectal tissues of the TCGA dataset. The dotted line indicates the threshold above which a malignant status would be predicted. TCGA: The Cancer Genome Atlas; COAD: colon cancer cohort from TCGA; READ: rectal cancer cohort from TCGA.

Since the source of the circulating miRNAs included in our liquid biopsy method may have its origin from colorectal cancer, we sought to investigate the four-miRNA model performance in predicting malignancy directly in tumors. Colorectal tumors (*n* = 615) and non-neoplastic samples (*n* = 11) from The Cancer Genome Atlas (TCGA) database were used in this approach. A high classification efficiency was obtained (99.5% accuracy, 99.7% sensitivity, 90.9% specificity, AUC = 0.998) by adapting the model threshold for RNA sequencing quantification (cancer prediction threshold score >19.14) and applying the same weight for each marker (Figure 5). 

After being tested on CRC samples from TCGA, the SVM model was also applied to 14 other tumor types from the Pan-Cancer cohort. Despite a relatively high discrimination power observed for urothelial bladder carcinoma (AUC = 0.878), the model was found to be CRC-specific (AUC = 0.998) (Figure S2).

### 2.5. Putative mRNA Targets and Pathways Regulated by the Selected miRNAs

**Figure 6:**
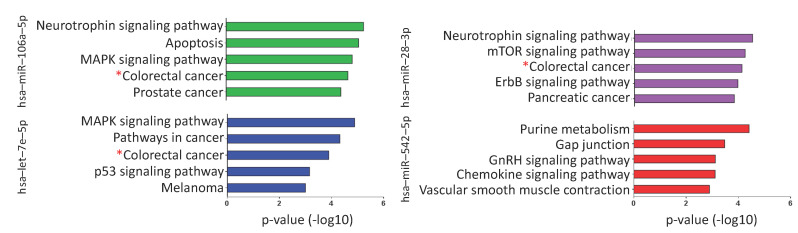
Biological pathways enriched with the mRNAs predicted to be targets of miR-106a-5p, let-7e-5p, miR-28-3p, and miR-542-5p. The colorectal cancer pathway (red star) is among the most significant pathways for three out of four tested miRNAs (miR-106a-5p, let-7e-5p, and miR-28-3p). *p*-Value expressed as −log_10_.

MicroRNAs regulate numerous target mRNAs that are involved in critical signaling pathways. Based on predicted interactions (miRWalk, miRanda, RNAhybrid, and Targetscan), miR-106a-5p, let-7e-5p, miR-28-3p, and miR-542 were estimated to regulate 2239, 1020, 637, and 203 mRNA targets, respectively (Table S6). The biological pathways enriched with the miRNA targets (performed separately for each miRNA) were mainly cancer-related (Tables S5 and S6 and Figure S3). The colorectal cancer pathway was among the most significant pathways for three of four tested miRNAs (miR-106a-5p, let-7e-5p, and miR-28-3p) (Figure 6).

## 3. Discussion

CRC screening methods include stool-based tests for occult blood search and endoscopic or radiologic imaging [29]. According to the updated National Comprehensive Cancer Network Clinical Practice Guidelines in Oncology, colonoscopy remains an effective and sensitive procedure for the detection of CRC compared with other screening modalities [29]. However, the limiting access to care, lack of adequate bowel preparation, bleeding, and colon perforation are among its complication risks [30]. New protocols have been described to overcome these limitations. The circulating Septin 9 methylated DNA demonstrated 73.3% sensitivity for CRC detection, comparable with that of the fecal immunochemical test (68.0%) [31], and is FDA-approved as an emerging, more accessible blood-based test option [29]. The performance of these tests is still far from ideal, and novel and sensitive blood biomarkers remain demanded. 

In the present study, the combination of four circulating overexpressed miRNAs (let-7e-5p, miR-106a-5p, miR-28-3p, and miR-542-5p) distinguished patients with CRC from healthy subjects and individuals with precursor lesions, particularly, hyperplastic polyps and adenomas. These findings indicate the potential of this circulating miRNA signature in predicting tumors in the colon and rectum at early stages. We used the Recursive Feature Elimination method to test multiple marker combinations and LOOCV to estimate the performance to avoid a marker selection bias. The combinations tested in our study were systematically defined, including only statistically significant individual markers overrepresented in the plasma of the cancer patients, using the recursive elimination method before training the classifier (SVM method). The recursive feature elimination method is based on removing the weakest features until a specific number of features is reached, avoiding collinearity and dependencies inside the model [32].

The four-miRNA-based signature discovered in our screening phase was tested in subjects at different colorectal carcinogenesis stages and validated in the cohort of colorectal samples from the TCGA database. The classifier designed to be a plasma miRNA signature also demonstrated high performance in differentiating cancer from non-cancer colorectal tissues, which infers the method’s accuracy. Strategies using biomarker signatures increase a method’s significance by boosting its diagnostic efficiency. Eslamizadeh et al. analyzed a panel of eight miRNAs to compare the plasma of CRC patients with that of healthy controls. Among the miRNAs investigated, four miRNAs distinguished these groups, but the diagnostic perspective was reduced by the independent analyses of each miRNA [21]. Other studies proposed plasma miRNA panels with potential clinical value for early CRC detection, demonstrating an AUC = 0.8356–0.866, with 78–91% sensitivity and 79–88% specificity, but not performing external validation of the miRNA panels in tumor tissues [33,34]. 

Considering that changes in miRNAs expression are expected during tumor development [35], a tumor signature essentially must be validated as a whole and bear the diagnostic power of their units combined. In line with that, Zanutto and colleagues proposed a plasma miRNA-based test associated with the fecal immunochemical test to identify patients that could benefit from subsequent colonoscopy [24]. The authors categorized miRNA signatures as specific for low-grade adenoma, high-grade adenoma, or cancerous lesions. Interestingly, they found increased expression of some miRNAs in high-grade adenomas but reduced expression of the same in low-grade adenomas and cancerous lesions [24]. Such an approach diverges from ours, since we propose identifying miRNAs that are progressively expressed along with the carcinogenic process. Our strategy also contrasts with other studies that grouped advanced adenoma and CRC and found plasma- or serum-derived miRNA signatures differentially expressed with respect to control individuals [22,36]. Grouping non-neoplastic lesions with neoplastic tumors limits the identification of markers that could differentiate these groups of lesions.

Another advantage of our proposed plasma miRNA signature compared to others previously reported is the superior classification performance when applied to tissue specimens [37,38]. In CRC samples compared with normal tissues, Zhu et al. (2017) reported a three-miRNA panel with good accuracy in predicting tumor samples (AUC = 0.830) [38]. Notably, our four-miR classifier presented a higher diagnostic efficiency (AUC = 0.998). Using stringent criteria for the selection of key miRNAs as described in our study increases the diagnostic potential of a given signature. The use of miRNA profiles in liquid biopsies of cancer patients has received special attention in recent years. However, the main message from studies in this area is the difficulty in generating reproducible data. The method and source (serum, total plasma, purified extracellular vesicles, for instance) used to isolate microRNAs can result in variations in the miRNA profile [39]. Remarkably, we tested the performance of the four-miR classifier in the GEO dataset. Among the studies evaluated, five of them validated our classifier model, despite being serum-based analyses. Therefore, the four-miR classifier proposed in our study is suitable to be used despite the blood collection method (serum or plasma). It is important to note that if the signature is effective both in plasma and in serum, samples included in the same study must be collected with the same protocol.

Among the miRNAs herein detected, let-7e-5p is broadly described in several cancers, including head and neck and rectum [25,40]. Interestingly, let-7e-5p was suggested as a prognostic marker for inducing metastatic capacity in rectal carcinomas [25]. In addition, let-7e-5p-inducing cell migration was further confirmed in the colon carcinoma-derived Caco-2 cell line transfected with hsa-let-7e-5p-carrying plasmids. The underlying metastatic mechanism is unclear but seems to involve the modulation of MYC pathways [25,41]. The second key miRNA identified in our diagnostic classifier was miR-106a-5p, which showed a high discriminative power between the groups. miR-106a-5p overexpression contributes to cell invasion and is associated with 5-fluorouracil resistance in colorectal cancer patients [42]. The tumorigenic mechanism might involve the inhibition of apoptotic pathways, as demonstrated in breast cancer cells [43]. A translational approach demonstrated that miR-106a-5p is overexpressed in colorectal cancer and associated with tumor stage, vascular invasion, and lymph node metastasis, reducing disease-free survival [44]. Similarly, miR-28-3p expression was also related to colon and rectum malignancies. miR-28 is described to induce tumor metastases in CRC animal models and increase the migration and invasiveness capacity of the colorectal cancer cell line HCT-116 [45]. 

We also found miR-542-5p overexpression in the plasma but with lower discriminating capacity in subjects with CRC than other miRNAs, including miR-28-3p and miR-106a-5p. One possible explanation might involve the reduced interaction between the signaling pathways that these miRNAs regulate. miR-542-5p is found to induce mitochondrial dysfunction and activation of SMAD2/3 phosphorylation [46], a signaling molecule downstream of transforming growth factor-β (TGF-β). TGF-β is a critical player in epithelial–mesenchymal transition, favoring tumor cell survival and dissemination [47]. Additionally, it allows tumor microenvironment remodeling to support cancer progression [47]. Together with TGF-β, dysfunctional mitochondria can trigger gene expression changes, altering cell morphology and function and resulting in a pro-tumorigenic phenotype [48]. 

Despite the experimental and clinical relevance, the specificity of single miRNAs as a diagnostic tool is limited since most miRNAs are expressed by other tumor types and inflammatory conditions, generating false-positive or false-negative results [49]. The use of a diagnostic classifier must then overcome an miRNA biological function. Consistently, the high efficiency of our four-miR-based diagnostic tool (99.5% accuracy, 99.7% sensitivity, 90.9% specificity for tumor specimens) suggests its technical reliability. 

However, our study has some limitations, including a low number of patients. In this setting, the signature proposed was not sensitive enough to discriminate subsets of patients either in the non-cancer or in the cancer groups. In addition to the possibility that some interesting miRNAs might be lost due to the limited number of cases used in our screening phase, a larger number of miRNAs were not tested. Current information on the human miRNome estimates about 2300 human mature miRNAs, only 50% (1115 miRNAs) of which are annotated in miRBase V22 [50]. Then, technical limitations must be considered regarding the number of miRNAs analyzed (381 miRNAs) in our study. Second, the TCGA database involves some restrictions, since the data are not curated, and several comorbidities might influence population variability. The validity of the proposed signature might also consider the simultaneous presence of other morbidities. The prognostic applicability also merits further investigation in prospective studies. In our protocol, blood samples were collected before either the endoscopic procedure or chemotherapy. This strategy was essential to prevent any bias associated with anesthetics and chemotherapy administration. After the initiation of chemotherapy, tumor biology changes as well as the miRNA profile [51]. The type of chemotherapeutic regimens (drugs, dose intensity, time, etc.) administered is also patient-specific, which increases the number of variables to control. Furthermore, miRNAs expression can be altered as a consequence of the treatment [52] and have a role in cancer drug resistance. Based on these statements, our study was designed to identify a signature useful as a diagnostic tool. 

Therefore, the combination of let-7e-5p, miR-106a-5p, mir-28-3p, and miR-542-5p as a proposed signature does not intend to replace the current gold standards in diagnostic measures but as a complementary tool to improve cancer-screening methods. The combined analysis of our four-miRNAs has potential clinical applicability and overwhelms the shortcomings of some circulating miRNA signatures that independently evaluate each of its composing miRNAs.

## 4. Materials and Methods 

### 4.1. Patients and Study Design

This observational, analytical, cross-sectional study was approved by the Ethics Committee of the involved institutions (Cancer Institute of Ceará, Haroldo Juaçaba Hospital; Walter Cantídio University Hospital, Federal University of Ceará—HUWC/UFC; and Dr. César Cals General Hospital-HGCC) (# 3.047.394, CAAE: 32361714.0.1001.5528). All subjects provided written informed consent, and the study was performed following the Declaration of Helsinki. 

The number of colorectal cancer patients admitted at the Oncology Department per year in the Cancer Institute of Ceará, Brazil is 200 individuals (N). The mean cycle quantification ($\bar{X}$) ± standard deviation(s) for the cancer patients recruited in the screening phase is 17.78 ± 1.92. Sample size calculation with a 95% confidence interval († = 2.306, considering t distribution having 8 degrees of freedom) and sampling error (e, 5% of the $\bar{X}$) was based on the following formula: *n* = [N × s^2^ × †^2^]/[(N − 1)×e^2^ + s^2^ × †^2^], indicating a sample size (*n*) of 16 per group (screening + discovery + validation sets) [53]. 

A total of 109 subjects were stratified into non-cancer (27 healthy controls, 17 individuals with hyperplastic polyps, and 24 with adenoma) and cancer patients (20 CRC and 21 metastatic CRC) (Table 1). All individuals were submitted to a routine colonoscopy; the suspected lesions, when identified, were processed for histopathological evaluation to confirm the diagnosis. Eligibility criteria for patient classification into groups also comprised: (1) healthy volunteers (absence of colorectal lesions), (2) hyperplastic polyps (larger than 10 mm, removed by a colonoscopic procedure—polypectomy), (3) adenomas (larger than 10 mm or with a high degree of dysplasia or at least 20% of the villous component removed with a colonoscopic procedure), (4) patients with advanced non-metastatic CRC (tumor lesions >1.0 cm) and (5) metastatic CRC (at advanced stages and distant CRC metastasis or post-surgical resection with active disease). Considering that chemotherapy alters the miRNA profile in tumor and te blood [51], the patients were included in this study before exposure to chemotherapy. All patients enrolled in this study were older than 18 years. 

The following exclusion criteria were adopted: clinical diagnosis of familial adenomatous polyposis or Lynch syndrome; the presence of more than 10 colorectal adenomas, inflammatory bowel disease, or diabetes; other primary tumors at the time of recruitment; chemotherapy or radiotherapy before blood collection; incomplete colonoscopy; inadequate preparation for colonoscopy; and the presence of any degree of hemolysis (determined by visual inspection) in plasma samples. The absence of hemolysis in samples that proceeded for analysis was later confirmed by the delta cycle quantification (Cq) (miR-23a and miR-451), positive if >7 [54]. 

The blood samples (8 mL) were collected in EDTA-K2 vials (BD Vacutainer^®^, Becton Dickinson, São Paulo, Brazil) by venous puncture. Plasma was obtained by sample centrifugation at 1000× *g* (4 °C for 10 min), transferred to cryotubes, and then stored at −80 °C until use. 

### 4.2. RNA Extraction and cDNA Synthesis

RNA was isolated from 1 mL of plasma samples using the TRIzol reagent (Life Technologies, Carlsbad, CA, USA) according to the manufacturer’s instructions, followed by purification with the columns from the miRNeasy Mini kit (Qiagen, Valencia, CA, USA). Samples triplicates were used for column saturation. RNA was eluted in nuclease-free water and treated to eliminate genomic DNA contamination with a DNA-free kit (Life Technologies, Carlsbad, CA, USA). Due to its low abundance, the RNA was quantified with the Bioanalyzer small RNA Analysis kit (Agilent Technologies, Santa Clara, CA, USA) and the Agilent 2100 Bioanalyzer (Agilent Technologies, Santa Clara, CA, USA). RNA (75 ng) and 4.5 μL of the Megaplex™ RT Primers, Human Pool A v2.1 (Thermo Fisher Scientific, Pleasanton, CA, USA) were used to obtain a final volume of 7.5 µL of reaction per sample for the synthesis of cDNA, according to the manufacturer’s recommendations. Once converted to cDNAs, these miRNAs were subjected to a pre-amplification step using the Megaplex^TM^ PreAmp Primers Human Pool A Kit and TaqMan^TM^ Master Mix (Applied Biosystems, Foster City, CA, USA). Then, the resulting pre-amplified product was used to detect miRNA expression by TLDA.

### 4.3. MicroRNA Relative Quantification by TaqMan Low-Density Array 

The miRNA expression analysis of 24 cases (discovery set) was performed using the TaqMan^TM^ Array Human MicroRNA A Cards v2.0 (TLDA) (Applied Biosystems, Foster City, CA, USA), composed of 377 miRNAs, three small-nucleolar RNAs, and one negative control (exogenous miRNA). The reactions were performed in the Biosystems Prism 7900HT Fast Real-Time PCR sequence detection System (Applied Biosystems, Foster City, CA, USA). Sequences with constant deficient expression were removed (*n* = 85), based on null cycle quantification (Cq) in more than 5% of the samples (after 40 cycles) of the cancer and non-cancer groups (Table S1). The miRNAs detected in at least 95% of the samples in any of the biological groups were further evaluated. RT-qPCR normalization was carried out following the Livak and Schmittgen method (2001) [55]. The arithmetic mean of the Ct/Cq values from the control samples for each miRNA was used as a calibrator value in the normalization. The geometric mean of the ΔCt from all filtered miRNAs (*n* = 292) was used as a reference quantification (normalization factor) and integrated with the 2^−ΔΔCt^ model to obtain the relative quantification of the target miRNAs. The log_2_ transformed values were further quantile-normalized using the program BRB ArrayTools v. 4.4.0 (Biometric Research Branch, National Cancer Institute) to avoid inter-sample variation. Hierarchical clustering analysis was implemented with one minus correlation distance and complete linkage (BRB ArrayTools). 

### 4.4. Circulating miRNA-Based Diagnostic Model

Non-cancer (5 healthy volunteers, 5 hyperplastic polyps, and 5 adenomas) and cancer samples (5 CRC and 4 metastatic CRC) were used to identify miRNAs differentially expressed in the screening phase. The screening phase was performed with a reduced number of samples per group; however, the number of samples was further enlarged in the validation set to confirm the importance of the selected miRNAs. The groups were statistically compared using a random variance t-test, adopting a *p*-value < 0.05 and FC (fold change) ≥|1.5|. The classifiers were trained by Support Vector Machine (BRB Array Tools), considering the higher circulating miRNAs in the cancer group. The RT-qPCR values used in the statistical analysis, illustrations, and introduced into the mathematical model were the log_2_-transformed after the normalization using the Livak method [55].

### 4.5. Validation of Selected microRNAs 

Since the use of hundreds of miRNAs to obtain a reference quantification would not be feasible for the normalization in the individual TaqMan assays (Discovery and Validation Sets), we used the best pair of endogenous control candidates based on the TLDA results. From the 292 filtered miRNAs in TLDA, 96 candidates detected at a high frequency in the plasma of healthy individuals and patients (mean Cq < 20) were evaluated by the Genorm software [56]. According to this analysis, miR-423-5p and miR-361-5p were the most stable miRNAs, presenting the average expression stability of 0.23 (after serial exclusion of the most variable candidates) and pairwise variation of 0.065. Therefore, these two miRNAs were subsequently used as endogenous references to normalize the target miRNAs in the individual TaqMan assays. Although miR-423-5p was previously described in lower levels in CRC than in the source tissue [57], a significant impact on the bloodstream levels would be more likely to be observed in miRNAs overexpressed in tumors [58].

TaqMan MicroRNA Individual assays (TaqMan™ Fast Advanced Master Mix—Life Technologies, Austin, TX, USA) were carried out using the AB7900 Real-Time System (Applied Biosystems). Selected circulating miRNAs found at higher levels in cancer cases (let-7e-5p, miR-106a-5p, miR-28-3p, and miR-542-5p) were further tested (Table 2). The expression levels of these four miRNAs were determined by RT-qPCR in the discovery set of cases (*n* = 22, including 5 healthy volunteers, 5 hyperplastic polyps, 5 adenomas, 5 CRC, and 2 metastatic CRC patients) and 85 additional cases (validation set) composed of 22 healthy volunteers, 12 hyperplastic polyps, 19 adenomas, 15 CRC, and 17 metastatic CRC patients. The reactions were performed in triplicates. Sample insufficiency accounted for a reduction in group size between the screening phase and the discovery set in the metastatic CRC group.

### 4.6. Cross-Study Validation of the Circulating miRNA Model

Publicly available databases were interrogated to test the circulating miRNA model using GEO datasets (https://www.ncbi.nlm.nih.gov/gds, accessed on 15 January 2021). The search included miRNA based-study types (expression profiling by RT-PCR, non-coding RNA profiling by array, non-coding RNA profiling by genome tiling array, and non-coding RNA profiling by high-throughput sequencing), with a minimum of 40 samples, using the following terms: (colorectal OR rectal OR colon OR bowel OR gut) AND (cancer OR carcinoma OR tumor OR tumor OR neoplasm OR malignant OR carcinoma) AND (miRNA OR microRNA OR small non-coding) AND (healthy OR control OR non-cancer) AND (serum OR blood OR plasma OR circulating OR “liquid biopsy” OR biofluid). The inclusion criteria were: (1) at least 20 CRC and 20 controls (healthy or non-cancer individuals); (2) datasets with published results. The exclusion criteria were: (1) duplicate data reported in other studies; (2) lack of clinical/histological information; (3) datasets related to letters, editorials, case reports, or case series (Figure 4A). This analysis was conducted according to the Preferred Reporting Items for Systematic Reviews and Meta-Analyses (PRISMA) [59].

### 4.7. Data Comparison with Colorectal Tissues from the TCGA Database

The diagnostic model performance of our four selected circulating miRNAs was additionally investigated in solid tumors from TCGA database. Normalized miRNA expression values for the TCGA Pan-Cancer cohort were downloaded from the UCSC Xena Browser (GDC Pan-Cancer, https://xenabrowser.net/ on 21 June 2020). Samples within this cohort were grouped by cancer type and used to test the diagnostic value of the four-miRNA diagnostic algorithm. Only tumor types with more than 50 primary tumors and 10 non-neoplastic tissues available were included. The CRC cohort of small RNA sequencing data from TCGA (colon = 453; rectum = 162) and non-neoplastic adjacent tissues (colon = 8; rectum = 3), and other tissue types were used to apply the SVM method (same weights applied for each marker), and the AUC was assessed without establishing a specific diagnostic cut-off.

### 4.8. Prediction of Genes Regulated by miRNAs and Pathway Enrichment Analysis

An in silico prediction of the potential mRNA targets from selected miRNAs was employed using the miRWalk 2.0 tool (http://www.umm.uni-heidelberg.de/apps/zmf/mirwalk/, accessed on 21 June 2020). Only interactions retrieved by four different and widely used algorithms (miRWalk, miRanda, RNAhybrid, and Targetscan) were considered. A pathway enrichment analysis (Kyoto Encyclopedia of Genes and Genomes, KEGG, database) was implemented using miRWalk 2.0 (*p*-value from hypergeometric test <0.01 and FDR calculated by Benjamini and Hochberg method). The pathway enrichment analysis was performed separately for each miRNA (sets of target mRNAs as input) using default parameters.

### 4.9. Statistical Analysis

The results were statistically evaluated using GraphPad Prism (v. 6.0; GraphPad Software Inc., La Jolla, CA, USA), IBM SPSS Statistics for Windows, Version 25.0 (IBM Corp. Armonk, NY, USA), and BRB Array Tools software were employed in the statistical analysis and illustrations. The area under the curve (AUC, ROC curves) was calculated in the GraphPad Prism software. The classification performance with 95% confidence intervals was estimated using the Vassarstats online calculator (http://www.vassarstats.net, accessed on 21 June 2020). Significant values were considered with *p*-value < 0.05.

## 5. Conclusions

In summary, a plasma miRNA signature (let-7e-5p, miR-106a-5p, and miR-28-3p) was identified with potential use as a minimally invasive procedure for the diagnosis of CRC. Its accuracy was confirmed by a cross-study validation step using independent publicly available datasets. These results indicate the importance of improving colorectal cancer diagnostic methods by identifying new biomarkers that can be used to complement standard procedures in clinical practice. Therefore, circulating miRNAs signatures are promising candidates as liquid biopsies of high significance for detecting the carcinogenic process in the colon and rectum.

## Figures and Tables

**Table 1:** Clinical–demographic characteristics.

Variables		Groups					
		Total		Non-Cancer		Cancer	
		*n*	%	*n*	%	*n*	%
Gender							
	Female	61	55.96%	40	65.57%	21	34.43%
	Male	48	44.04%	28	58.34%	20	41.66%
Age							
	Up to 60 years	59	54.12%	40	67.79%	19	32.21%
	>60 years	50	45.88%	28	56%	22	44%
Smoking status							
	Never smoker	72	66.06%	49	68.05%	23	31.94%
	Former smoker	25	22.93%	13	52%	12	48%
	Current smoker	12	11.01%	6	50%	6	50%
Medication							
	No	59	54.12%	40	68%	19	32%
	Yes	50	45.88%	28	56%	22	44%

**Table 2:** Classification performance of the four-miR-based classifier used to distinguish colorectal cancer from non-cancer individuals.

Metric	TLDA Assay	Single Assays	
	Screening Phase	Discovery Set	Validation Set
	Estimate (CI_95%_)	Estimate (CI_95%_)	Estimate (CI_95%_)
Sensitivity	88.9 (50.7–99.4)	57.1 (20.2–88.2)	59.4 (40.8–75.8)
Specificity	86.7 (58.4–97.7)	80.0 (51.4–94.7)	86.8 (74–94.1)
PPV	80.0 (44.2–96.5)	57.1 (20.2–88.2)	73.1 (51.9–87.6)
NPV	92.9 (64.2–99.6)	80 (51.4–94.7)	78.0 (64.9–87.3)
AUC	0.867 (0.710–1.000)	0.743 (0.501–0.985)	0.716 (0.600–0.832)

CI_95%_: 95% confidence interval; PPV = positive predictive value; NPV = negative predictive value.

## Data Availability

The data presented in this study are openly available in this manuscript.

## Supplementary Materials

The following are available online at https://www.mdpi.com/2072-6694/13/7/1493/s1. Figure S1: The area under the ROC curves from the four plasma miRNA markers individually and combined. Figure S2: Performance of the 4-miRNA classifier in different types of solid tumors from TCGA (Pan-Cancer cohort). Figure S3: Network of interaction between miRNAs and mRNAs. Table S1: Assay target sequence and miRNA detection criteria. Table S2: Circulating miRNAs identified in cancer vs. non-cancer cases. Table S3: Mature miRNA sequences. Table S4: GEO datasets used in the cross-study validation analysis. Table S5: Significant pathways (*p*-value < 0.01) enriched with the predicted target genes of miRNAs included in the diagnostic model. Table S6: Biological target mRNAs enriched with the miRNA targets (performed separately for each miRNA).
