# Supplementary material for: Circulating let-7e-5p, miR-106a-5p, miR-28-3p, and miR-542-5p as a Promising microRNA Signature for the Detection of Colorectal Cancer
Source: Cancers (Basel). 2021 Mar 24;13(7):1493. doi: 10.3390/cancers13071493 (PMC8037203; doi:10.3390/cancers13071493)
Supplement: Supplementary file 1 [file cancers-13-01493-s001.zip › cancers-1123258-supple/cancers-1124258-crosscheck-SUPPL-1.docx]

Supplementary Materials: Circulating let-7e-5p, miR-106a-5p, miR-28-3p, and miR-542-5p as a Promising microRNA
Signature for the Early Detection of Colorectal Cancer

Camila Meirelles S. Silva, Mateus C. Barros-Filho, Deysi Viviana T. Wong, Julia Bette H. Mello,
Livia Maria S. Nobre, Carlos Wagner S. Wanderley, Larisse T. Lucetti, Heitor A. Muniz, Igor Kenned D. Paiva, Hellen Kuasne, Daniel Paula P. Ferreira, Maria Perpétuo S. S. Cunha, Carlos G. Hirth, Paulo Goberlânio B. Silva, Rosane O. Sant’Ana, Marcellus Henrique L.P. Souza, Josiane S. Quetz, Silvia R. Rogatto and
Roberto César P. Lima-Junior


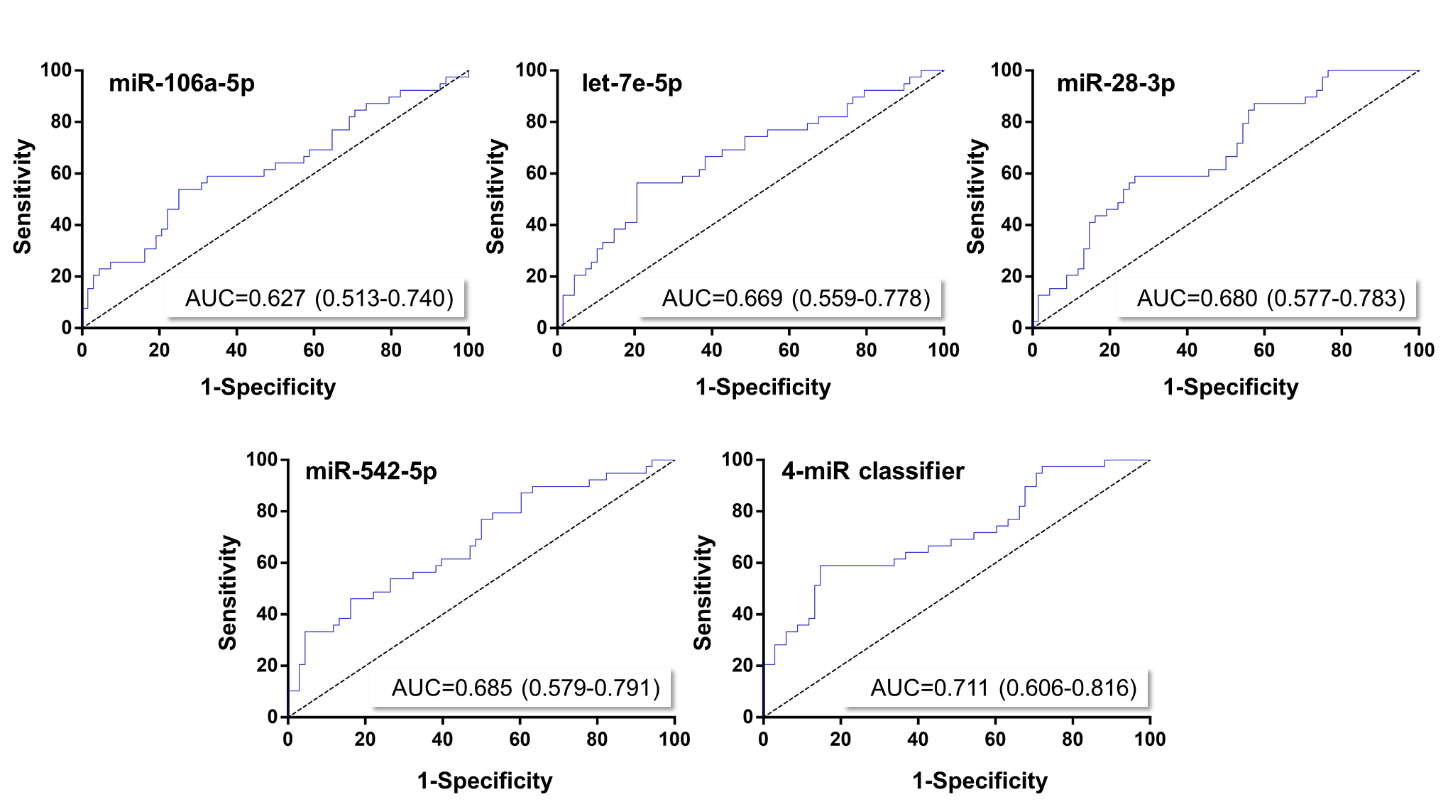


**Figure S1.** The area under the ROC curves of the four plasma miRNA markers individually and combined. AUC: area under the ROC curve (95% confidence interval).


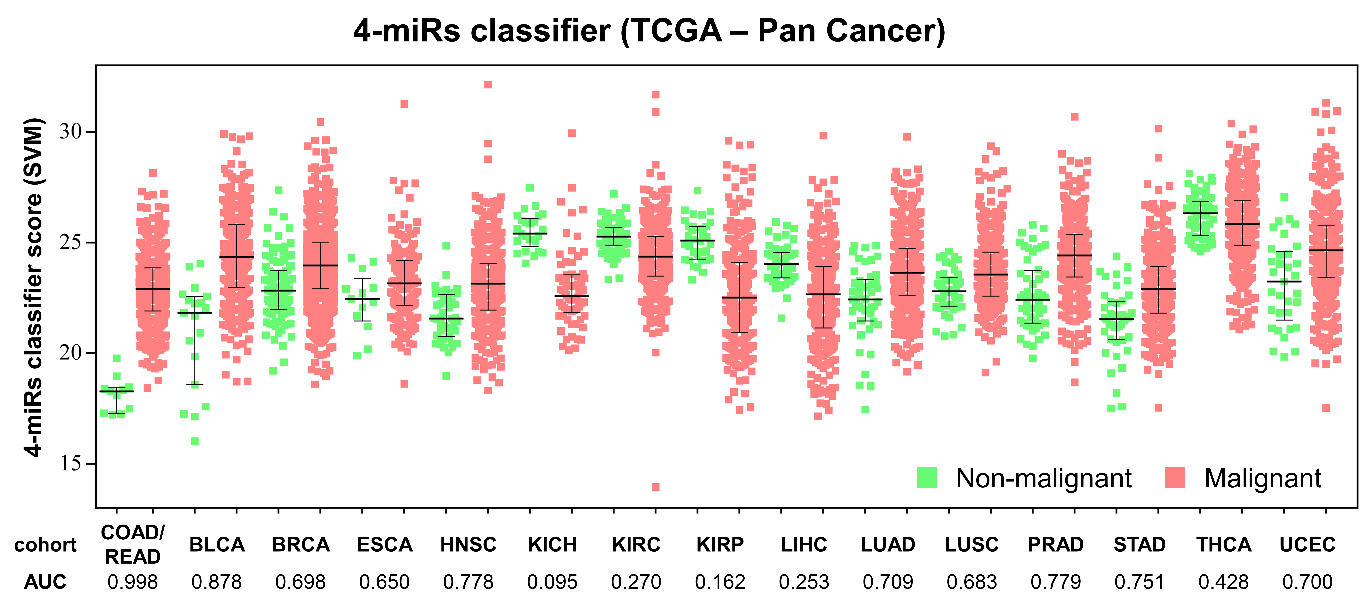


**Figure S2.** Performance of the 4-miRNA classifier in different types of solid tumors from TCGA (Pan-Cancer cohort). Distributions of the SVM scores for the non-malignant and malignant samples within the TCGA Pan-Cancer cohort. Error bars indicate the median score and the interquartile range; SVM= Support Vector Machines; AUC = area under the receiver operating characteristic curve; COAD= Colon adenocarcinoma; READ= Rectum adenocarcinoma; BLCA= Bladder Urothelial Carcinoma; BRCA= Breast invasive carcinoma; ESCA= Esophageal carcinoma; HNSC= Head and Neck squamous cell carcinoma; KICH= Kidney Chromophobe; KIRC= Kidney renal clear cell carcinoma; KIRP= Kidney renal papillary cell carcinoma; LIHC= Liver hepatocellular carcinoma; LUAD= Lung adenocarcinoma; LUSC= Lung squamous cell carcinoma; PRAD= Prostate adenocarcinoma; STAD= Stomach adenocarcinoma; THCA= Thyroid carcinoma; UCEC= Uterine Corpus Endometrial Carcinoma.


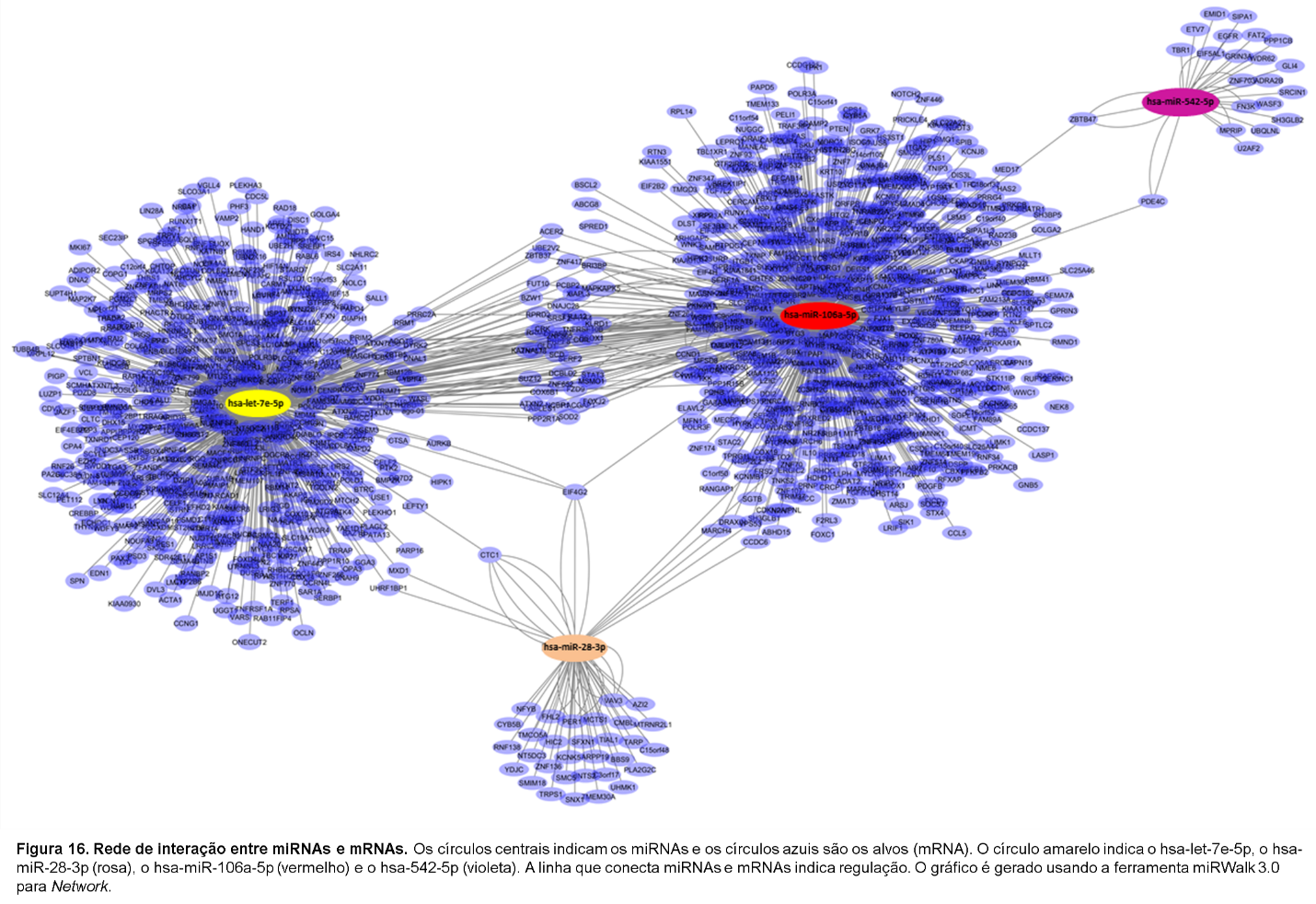


**Figure S3.** Network of interaction between miRNAs and mRNAs. The central circles represent the miRNAs, and the blue circles are the targets (mRNAs). The circles indicate hsa-let-7e-5p (yellow), hsa-miR-28-3p (pink), hsa-miR-106a-5p (red), and hsa-miR-542-5p (violet). The line connecting the miRNA to the mRNA indicates the regulator. The network was generated using the miRWalk 3.0 tool.

**Table S2.** Circulating miRNAs identified in cancer vs. non-cancer cases.

| **microRNA ID** | ***p*-Value** | **FDR** | **FC** |
| --- | --- | --- | --- |
| hsa-miR-106a-5p | 0.003 | 0.664 | 1.50 |
| hsa-let-7e-5p | 0.011 | 0.664 | 1.60 |
| hsa-miR-454-3p | 0.015 | 0.664 | 1.66 |
| hsa-miR-203a | 0.016 | 0.664 | 1.97 |
| hsa-miR-28-3p | 0.021 | 0.664 | 1.65 |
| hsa-miR-542-5p | 0.022 | 0.664 | 1.79 |
| hsa-miR-519a-3p | 0.033 | 0.664 | −1.65 |
| hsa-miR-190a-5p | 0.037 | 0.664 | −1.76 |
| hsa-miR-383-5p | 0.045 | 0.664 | −5.39 |

*p*-value: random variance *t* test; FDR: False Discovery Rate; FC: Fold Change (Cancer/Non-cancer).

**Table S3.** Mature miRNA sequences.

| **miRNA** | **Identification Code** | **Access Number** | **Mature miRNA Sequence** |
| --- | --- | --- | --- |
| hsa-let-7e-5p | 478579_mir | MI0000066 | UGAGGUAGGAGGUUGUAUAGUU |
| hsa-miR-106a-5p | 478225_mir | MI0000113 | AAAAGUGCUUACAGUGCAGGUAG |
| hsa-miR-28-3p | 477999_mir | MI0000086 | CACUAGAUUGUGAGCUCCUGGA |
| hsa-miR-542-5p | 478337_mir | MI0003686 | UCGGGGAUCAUCAUGUCACGAGA |
| hsa-miR-423-5p | 478090_mir | MI0001445 | UGAGGGGCAGAGAGCGAGACUUU |
| hsa-miR-361-5p | 478056_mir | MI0000760 | UUAUCAGAAUCUCCAGGGGUAC |

**Table S4.** GEO datasets used in the cross-study validation analysis.

| **Study ID** | **Sample Type** | **Platform** | **Samples** | | **Publication (PMID)** | **Country** |
| --- | --- | --- | --- | --- | --- | --- |
|  |  |  | **CRC** | **Controls** |  |  |
| GSE106817* | serum | microarray | 115 | 2759 | 30333487 | Japan |
| GSE113740* | serum | microarray | 25 | 969 | 32025611 | Japan |
| GSE112264* | serum | microarray | 50 | 41 | 30808771 | Japan |
| GSE124158* | serum | microarray | 30 | 275 | 30898996 | Japan |
| GSE113486* | serum | microarray | 40 | 100 | 30382619 | Japan |
| GSE59856* | serum | microarray | 50 | 150 | 25706130 | Japan |
| GSE71008* | plasma extracellular vesicles | high-throughput sequencing | 100 | 50 | 26786760 | USA |
| GSE25609* | plasma | microarray | 21 | 20 | 23267864 | Spain |
| GSE16256 | culture cells | high-throughput sequencing | 0 | 0 | 19829295; 20452322; 20944595; 21289626; 26030523; 28637928; 23526891 | USA |
| GSE34200 | cell lines and solid tissues | microarray | 0 | 0 | 23117585 | USA |
| GSE85589 | serum | microarray | 5 | 19 | - | South Korea |
| GSE40247^#^ | serum extracellular vesicles | microarray | 88 | 11 | 24705249 | Japan |
| GSE39833^#^ | serum extracellular vesicles | microarray | 88 | 11 | 24705249 | Japan |
| GSE41526 | plasma | microarray | 5 | 20 | 23472110 | USA |
| GSE138092 | cerebrospinal fluid | microarray | 2 | 0 | - | South Korea |
| GSE67075 | plasma | RT-qPCR | 16 | 8 | 25924769 | Ireland |

* Studies included in the meta-analysis; Underlined: originated from the same research group; ^#^ Replicated data.

**Table S5.** Significant pathways *(p*-value< 0.01) enriched with the predicted target genes of miRNAs included in the diagnostic model.

| **miRNA** | **KEGG Pathway ID (Number of Targets in the Pathway)** | ***p*-Value** | ***p*-Adjusted** |
| --- | --- | --- | --- |
| let-7e-5p | MAPK signaling pathway (32) | 1 × 10^−5^ | 0.002 |
|  | Pathways in cancer (35) | 5 × 10^−5^ | 0.008 |
|  | Colorectal cancer (14) | 1 × 10^−4^ | 0.020 |
|  | p53 signaling pathway (11) | 7 × 10^−4^ | 0.110 |
|  | Melanoma (11) | 1 × 10^−3^ | 0.158 |
|  | Pancreatic cancer (11) | 2 × 10^−3^ | 0.242 |
|  | Chronic myeloid leukemia (11) | 2 × 10^−3^ | 0.242 |
|  | Glioma (10) | 2 × 10^−3^ | 0.268 |
|  | Apoptosis (11) | 5 × 10^−3^ | 0.705 |
|  | Adherens junction (10) | 6 × 10^−3^ | 0.753 |
|  | Cytokine cytokine receptor interaction (24) | 6 × 10^−3^ | 0.772 |
|  | Prostate cancer (11) | 6 × 10^−3^ | 0.798 |
|  | Bladder cancer (7) | 6 × 10^−3^ | 0.798 |
|  | Heparan sulfate biosynthesis (5) | 1 × 10^−2^ | 0.986 |
|  | Adipocytokine signaling pathway (9) | 1 × 10^−2^ | 0.986 |
| miR-106a-5p | Neurotrophin signaling pathway (33) | 5 × 10^−6^ | 0.001 |
|  | Apoptosis (25) | 8 × 10^−6^ | 0.001 |
|  | MAPK signaling pathway (55) | 1 × 10^−5^ | 0.002 |
|  | Colorectal cancer (24) | 2 × 10^−5^ | 0.004 |
|  | Prostate cancer (24) | 4 × 10^−5^ | 0.007 |
|  | Non-small cell lung cancer (17) | 6 × 10^−5^ | 0.011 |
|  | Pathways in cancer (60) | 1 × 10^−4^ | 0.025 |
|  | Pancreatic cancer (20) | 2 × 10^−4^ | 0.033 |
|  | Glioma (18) | 2 × 10^−4^ | 0.040 |
|  | Long term potentiation (19) | 3 × 10^−4^ | 0.044 |
|  | Melanoma (19) | 3 × 10^−4^ | 0.044 |
|  | Endometrial cancer (15) | 5 × 10^−4^ | 0.079 |
|  | mTOR signaling pathway (15) | 6 × 10^−4^ | 0.099 |
|  | Progesterone mediated oocyte maturation (21) | 7 × 10^−4^ | 0.114 |
|  | Calcium signaling pathway (35) | 8 × 10^−4^ | 0.131 |
|  | Endocytosis (36) | 1 × 10^−3^ | 0.164 |
|  | Focal adhesion (38) | 1 × 10^−3^ | 0.206 |
|  | Wnt signaling pathway (30) | 2 × 10^−3^ | 0.271 |
|  | Renal cell carcinoma (17) | 2 × 10^−3^ | 0.324 |
|  | Small cell lung cancer (19) | 2 × 10^−3^ | 0.369 |
|  | Adipocytokine signaling pathway (16) | 4 × 10^−3^ | 0.674 |
|  | Gap junction (19) | 5 × 10^−3^ | 0.781 |
|  | T cell receptor signaling pathway (22) | 6 × 10^−3^ | 0.828 |
|  | GnRH signaling pathway (21) | 7 × 10^−3^ | 0.957 |
|  | TGF beta signaling pathway (18) | 7 × 10^−3^ | 1.000 |
|  | Bladder cancer (11) | 7 × 10^−3^ | 1.000 |
|  | p53 signaling pathway (15) | 8 × 10^−3^ | 1.000 |
|  | Ubiquitin mediated proteolysis (25) | 8 × 10^−3^ | 1.000 |
|  | Regulation of actin cytoskeleton (36) | 9 × 10^−3^ | 1.000 |
|  | Chronic myeloid leukemia (16) | 9 × 10^−3^ | 1.000 |
| miR-28-3p | Neurotrophin signaling pathway (12) | 1 × 10^−3^ | 0.127 |
|  | mTOR signaling pathway (7) | 1 × 10^−3^ | 0.194 |
|  | Colorectal cancer (9) | 2 × 10^−3^ | 0.232 |
|  | ErbB signaling pathway (9) | 2 × 10^−3^ | 0.291 |
|  | Pancreatic cancer (8) | 3 × 10^−3^ | 0.357 |
|  | Chronic myeloid leukemia (8) | 3 × 10^−3^ | 0.357 |
|  | Protein export (4) | 6 × 10^−3^ | 0.691 |
|  | TGF beta signaling pathway (8) | 7 × 10^−3^ | 0.754 |
|  | MAPK signaling pathway (17) | 7 × 10^−3^ | 0.798 |
|  | Progesterone mediated oocyte maturation (8) | 8 × 10^−3^ | 0.803 |
|  | Non-small cell lung cancer (6) | 8 × 10^−3^ | 0.810 |
| miR-542-5p | Purine metabolism (9) | 4 × 10^−5^ | 0.004 |
|  | Gap junction (6) | 3 × 10^−4^ | 0.033 |
|  | GnRH signaling pathway (6) | 8 × 10^−4^ | 0.073 |
|  | Chemokine signaling pathway (8) | 8 × 10^−4^ | 0.074 |
|  | Vascular smooth muscle contraction (6) | 1 × 10^−3^ | 0.120 |
|  | Progesterone mediated oocyte maturation (5) | 2 × 10^−3^ | 0.197 |
|  | Calcium signaling pathway (7) | 2 × 10^−3^ | 0.226 |
|  | Dilated cardiomyopathy (5) | 3 × 10^−3^ | 0.255 |
|  | Melanogenesis (5) | 4 × 10^−3^ | 0.343 |
|  | Oocyte meiosis (5) | 6 × 10^−3^ | 0.482 |
|  | Melanoma (4) | 6 × 10^−3^ | 0.487 |
|  | SNARE interactions in vesicular transport (3) | 7 × 10^−3^ | 0.562 |

*p*-value: hypergeometric test; *p* adj: *p* value adjusted by Benjamini and Hochberg method.
